# Isolated right ventricular thrombus in an adult patient with nephrotic syndrome: a case report

**DOI:** 10.1186/s13256-017-1491-0

**Published:** 2017-11-04

**Authors:** Severin Lempp, Vedat Schwenger

**Affiliations:** Clinic for Kidney, Hypertension and Autoimmune Diseases, Transplant Center Stuttgart, Klinikum Stuttgart, Kriegsbergstraße 60, 70174 Stuttgart, Germany

**Keywords:** Thrombus, Cardiac mass, Right ventricle, Nephrotic syndrome

## Abstract

**Background:**

Venous thrombosis in nephrotic syndrome is a well-described phenomenon. We report a case of an adult patient with an isolated thrombus in the right ventricle due to nephrotic syndrome, which was initially suspected to be a myxoma.

**Case presentation:**

A 28-year-old white woman presented to our emergency department with signs of fluid overload. On further evaluation, a right ventricular mass was detected, which was resected and was found to be a thrombus. No other manifestations of venous thrombosis were found. Further evaluation of the patient revealed a nephrotic syndrome, which caused augmented coagulopathy.

**Conclusions:**

We present a case of a patient in whom a right ventricular mass was the first sign of a renally derived coagulopathy. To the best of our knowledge, this is the first report of an isolated thrombus in the right ventricle due to nephrotic syndrome in an adult. In cases of isolated cardiac thrombi in adults, a further search for renal disease might be helpful to reveal the underlying cause.

## Background

Venous thrombosis in nephrotic syndrome is a well-described phenomenon that occurs in up to 60% of patients with nephrotic syndrome, mostly in the renal vein or in the deeper veins of the lower limb [1, 2]. The development of a hypercoagulable state in patients with nephrotic syndrome develops for multifactorial reasons, such as urinary loss of protein C, protein S, and antithrombin III, together with an increase in the production of procoagulatory factors. Further players are an alteration in platelet function, hypoalbuminemia, increased blood viscosity, and thrombocytosis [3]. In patients with renal failure, thrombi in the right ventricle can occur (e.g., as a consequence of a central venous line), whereas isolated thrombotic complications in the heart in nephrotic patients are described only in children [4].

**Fig. 1:**
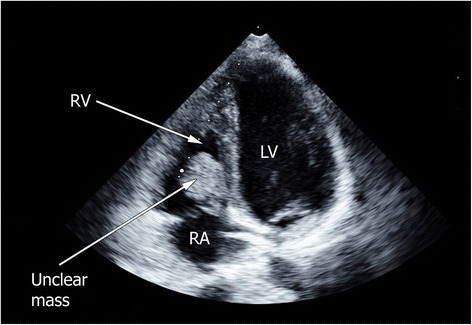
Right ventricular mass. Echocardiographic image of the thrombus in the right ventricle. *LV* Left ventricle, *RA* Right atrium, *RV* Right ventricle

Thrombosis in patients with nephrotic syndrome is quite common, but in adults, to our knowledge, an isolated thrombus of the right ventricle has not yet been described in the literature. Therefore, urinalysis in patients with cardiac thrombus of unknown origin might give a clue to the underlying cause.

## Case presentation

A 28-year-old white woman presented to our emergency department with dyspnea, edema of the lower limbs, and weight gain of 11 kg in the last 2 weeks. Her respiratory frequency was 20 breaths/minute, her heart rate was 82 beats/minute, her blood pressure was 98/65 mmHg, and her oxygen saturation was 100% on room air. Her body temperature was 36.7 °C.

The patient was alert and cooperative; in no acute distress; and orientated to person, place, and time. An examination of her lungs revealed clear auscultation and percussion without rales, wheezing, or diminished breath sounds. A cardiac examination revealed normal S1 and S2 and no S3, S4, or murmurs; the rhythm was regular. She had no cyanosis or pallor. Her extremities were warm and well perfused with distinctive edema of the lower limbs. Her strength and sensation were symmetric and intact throughout. Her abdomen had positive bowel sounds and was soft, nondistended, and nontender.

The patient’s white blood cell count was 9600/μl, and her C-reactive protein level was 0.3 mg/dl. Her serum creatinine was within the normal range (0.9 mg/dl). On further evaluation, her serum albumin was decreased to 8.4 g/L, and urinalysis revealed proteinuria of 16 g/g creatinine.

The patient was not taking any medication at the time of presentation. She was a housewife and had one son.

For further evaluation, echocardiography was performed, revealing a 3×3-cm mass in the right ventricle, which was suspected to be a myxoma (Fig. 1). Therefore, cardiac surgeons extracted the mass on the same day by using a minimally invasive technique. Histological examination yielded a relatively fresh thrombus and no signs of atypical cells or malignancy. Deep vein thrombosis of the legs could be excluded by color Doppler sonography. The patient had no clinical or echocardiographic signs of pulmonary embolism.

After discussing the urine findings with the patient, she reported an episode of proteinuria at the age of 7 years and again at 13 years, both of which were treated with steroids and chlorambucil. A kidney biopsy was not performed in childhood.

Because we suspected a very late recurrence of minimal change glomerulonephritis, and owing to the patient’s concomitant anticoagulation therapy, which was started after the surgery because of nephrotic syndrome, a kidney biopsy was again not performed. Treatment with oral steroids (1 mg/kg body weight) was immediately initiated. The patient’s proteinuria disappeared within 4 weeks, her weight went back to normal, and her serum albumin level 6 weeks later was also normal (35.9 g/L).

## Discussion

In this report, we present a case of a white woman with an isolated right ventricular mass. Most cardiac tumors are benign, with the majority of them being myxomas [5], which are more likely to occur in females than in males [6, 7]. Because of the echocardiographic findings in our patient, myxoma was the suspected diagnosis. Owing to the possible complications of this entity, prompt surgical removal was performed. Histologically, the mass was found to be a thrombus. Ultrasound was performed, which revealed no thrombosis of the lower extremities or in the renal veins.

On further evaluation, we diagnosed proteinuria within the nephrotic range. As a consequence of loss of anticoagulatory factors such as antithrombin III, venous thrombi are a common problem in these patients [3]. Arterial and venous thrombotic complications in patients with nephrotic syndrome are well known and occur in up to 60% of these patients [1]. The most common sites of these complications are the renal vein in up to 60%, and in the deep veins of the lower limb, they can occur in over 40%; pulmonary embolism has also been described [1, 8]. For this reason, anticoagulation therapy is initiated if the serum albumin level is < 20 g/L. Although there are several known cases of thrombotic complications of the heart, they usually occur together with other sites of thrombosis in patients with nephrotic syndrome [9]. Nevertheless, thrombus formation in or close to the right side of the heart is well known in nephrology departments. However, those thrombi are usually due to chronic dialysis catheters [10]. In one study, central vein thrombosis occurred in 28% of patients with a dialysis catheter [11]. These thrombi are related to the implantation of foreign material, as well as to patient factors, whereas right ventricular thrombus without a central venous line is rare. Isolated cardiac thrombi are described in children with nephrotic syndrome [4]. However, to the best of our knowledge, this is the first reported case in an adult.

## Conclusions

An isolated thrombus in the right ventricle without a previous central venous line or dialysis catheter is a very rare clinical condition. To the best of our knowledge, this is the first report where an isolated thrombus in the right ventricle was the only and leading finding in a renally derived coagulopathy in an adult with nephrotic syndrome. In patients with cardiac thrombi of unknown origin, urinary examination should be performed to diagnose or exclude nephrotic proteinuria.
